# Optimizing patient selection for stereotactic ablative radiotherapy in patients with locally advanced pancreatic cancer after initial chemotherapy - a single center prospective cohort

**DOI:** 10.3389/fonc.2023.1149961

**Published:** 2023-05-31

**Authors:** D. Doppenberg, F. J. Lagerwaard, S. van Dieren, M. R. Meijerink, J. J. van der Vliet, M. G. Besselink, G. van Tienhoven, E. Versteijne, B. J. Slotman, J. W. Wilmink, G. Kazemier, A. M. E. Bruynzeel

**Affiliations:** ^1^ Amsterdam UMC, Department of Radiation Oncology, Vrije Universiteit Amsterdam, Amsterdam, Netherlands; ^2^ Cancer Center Amsterdam, Amsterdam, Netherlands; ^3^ Amsterdam UMC, Department of Surgery, University of Amsterdam, Amsterdam, Netherlands; ^4^ Amsterdam UMC, Department Intervention Radiology, Vrije Universiteit Amsterdam, Amsterdam, Netherlands; ^5^ Amsterdam UMC, Department of Medical Oncology, Vrije Universiteit Amsterdam, Amsterdam, Netherlands; ^6^ LAVA Therapeutics, Utrecht, Netherlands; ^7^ Amsterdam UMC, Department of Medical Oncology, University of Amsterdam, Amsterdam, Netherlands; ^8^ Amsterdam UMC, Department of Surgery, Vrije Universiteit Amsterdam, Amsterdam, Netherlands

**Keywords:** pancreatic cancer, LAPC, radiotherapy, SABR, MRgRT, patient selection

## Abstract

**Background:**

The role of stereotactic ablative radiation therapy (SABR) as local treatment option after chemotherapy for locally advanced pancreatic cancer (LAPC) is evolving. However adequate patient selection criteria for SABR in patients with LAPC are lacking.

**Methods:**

A prospective institutional database collected data of patients with LAPC treated with chemotherapy, mainly FOLFIRINOX, followed by SABR, which was delivered using magnetic resonance guided radiotherapy, 40 Gy in 5 fractions within two weeks. Primary endpoint was overall survival (OS). Cox regression analyses were performed to identify predictors for OS.

**Results:**

Overall, 74 patients were included, median age 66 years, 45.9% had a KPS score of ≥90. Median OS was 19.6 months from diagnosis and 12.1 months from start of SABR. Local control was 90% at one year. Multivariable Cox regression analyses identified KPS ≥90, age <70, and absence of pain prior to SABR as independent favorable predictors for OS. The rate of grade ≥3 fatigue and late gastro-intestinal toxicity was 2.7%.

**Conclusions:**

SABR is a well-tolerated treatment in patients with unresectable LAPC following chemotherapy, with better outcomes when applied in patients with higher performance score, age <70 years and absence of pain. Future randomized trials will have to confirm these findings.

## Background

Pancreatic ductal adenocarcinoma has a dismal prognosis. At diagnosis, approximately half of the patients have metastasized disease and at least one third of all patients is diagnosed with a non-metastatic, locally unresectable tumor: locally advanced pancreatic cancer (LAPC) (1). A small percentage of patients with LAPC may become eligible for resection following induction chemotherapy, however 85% remains locally unresectable (2). Treatment of these patients focusses on local control, prolongation of life and preservation of quality of life, in which single or multi-regimen systemic chemotherapy plays an important role (3, 4). Based on extrapolation from randomized controlled trials (RCTs) in patients with metastatic PDAC, the current National Comprehensive Cancer Network (NCCN) guideline for LAPC recommends (modified) FOLFIRINOX (a combination of leucovorin, fluorouracil, irinotecan and oxaliplatin) for patients with a good performance score (PS) and a combination of gemcitabine and nab paclitaxel for patients with a poorer Karnofsky performance score (KPS) (5, 6). Patients who do not develop metastases during their systemic treatment may benefit from radiotherapy to delay local progression. A review that reports on overall survival (OS) in patients with LAPC treated with FOLFIRINOX, describes that almost two-third of patients received subsequent radiotherapy or chemoradiotherapy (7). Stereotactic ablative body radiation therapy (SABR) has a number of advantages over conventional radiotherapy and is nowadays recognized as a standard-of-care option in the treatment of LAPC in several guidelines (5, 8–10). SABR allows high-precision high-dose delivery in only few fractions whilst avoiding surrounding radio-sensitive organs at risk (OARs) (11). As a result, SABR causes limited radiation induced toxicity and thus allows quick resumption of systemic therapy, if indicated (11, 12). However, international consensus regarding the role and timing of SABR in the treatment of LAPC is lacking, as well as patient selection criteria. In order to establish such patient selection parameters for SABR, this study analyzed outcomes in LAPC patients treated with upfront chemotherapy followed by SABR.

## Materials and methods

### Study design

Clinical and outcome data of patients with unresectable LAPC after chemotherapy followed by SABR between June 2016 and March 2022 were selected from a prospectively maintained, ethics committee approved, institutional database.

### Study procedures

Patients were referred to the department of Radiation Oncology after a diagnosis of unresectable LAPC by consensus of a multidisciplinary tumor board. Patient characteristics prior to SABR collected in the database were age, gender, patient fitness scored as KPS (13), location of the tumor within the pancreas, use and duration of chemotherapy.

At our center SABR is delivered in the form of magnetic-resonance guided radiotherapy (MRgRT) aiming for a total dose of 40Gy in 5 fractions with dose escalation within the tumor. The objectives for target coverage were a V95% of the GTV ≥90% and a D2% up to 125% of the prescribed dose. Simulation imaging consisted of an Magnetic Resonance (MR)- and Computerized Tomography (CT) scan, both in supine position in shallow inspiration breath-hold. The gross tumor volume (GTV) is delineated on the simulation MR scan aided by diagnostic imaging in collaboration with a gastro-intestinal intervention radiologist. The GTV includes the tumor in the pancreas and any adjacent suspicious lymph nodes. No additional margin for microscopic tumor extension was applied for SABR. The planning target volume (PTV) was generated by the addition of a 3 mm margin around the GTV. The duodenum, stomach, bowel, liver, kidneys, and spinal cord were contoured as OARs. Maximum dose limits to the OARs (duodenum, bowel and stomach) were prioritized over target coverage (14). Radiation was delivered using respiratory gating during subsequent breath-hold periods in shallow inspiration. In addition to auditory feedback provided during treatment, gating of the tumor is augmented by visual feedback which is performed with the aid of an in-room MR compatible monitor, showing the actual tumor motion on a sagittal cine-MR. Daily adaptive planning is our standard approach for MRgRT of pancreatic cancer patients, which consists of MR imaging and recontouring of the target volume and relevant organs at risk within 2 cm distance, followed by online radiation plan re-optimization. As described for the pretreatment planning, organs-at-risk constraints are prioritized above target coverage for each fraction. Detailed information about the high-dose OAR constraints and the adaptive workflow used for daily plan adaptation in this patient group, is described in our earlier work (14). Patients received dietary instructions as treatment was delivered after 2 hours fasting. It was standard to prescribe prophylactic ondansetron prior to each fraction.

### Outcomes

Primary end point was overall survival (OS) defined as 1) time between date of diagnosis and date of death (of any cause) and 2) time between start date of SABR and date of death (of any cause). The secondary end points were local control rates according to RECIST criteria and toxicity (i.e. pain, nausea, diarrhea, fatigue) using the NCI-CTCAE toxicity criteria (version 5.0) (15, 16). Toxicity outcomes were collected both prior to and after SABR to assess the effect of SABR on these measures. The toxicity of all separate symptoms was corrected for the baseline absence or presence and severity. An increase as well as new occurrence of toxicity was noted as toxicity caused by SABR. Outcomes were collected at 6 weeks after SABR for ‘acute’ toxicity and during follow-up for ‘late’ toxicity.

### Statistical aspects/analyses

Data were analyzed using IBM SPSS Statistics for Windows version 26.0 (IBM Corp., Orchard Road Armonk, New York, NY). Categorical data are presented as percentages and frequencies. Normally distributed continuous data are presented as means and standard deviations (SDs). Primary analyses consisted of OS assessment using Kaplan-Meier estimations from the date of diagnosis and start date of treatment with SABR until the date of death or last moment of follow-up. As data was prospectively collected from start of SABR, stratified Kaplan-Meier analyses were performed among subgroups on OS from start of MRgRT. Continuous variables were divided in subgroups based on the median. Subgroups consisted of high versus low age (≤70 years versus >70 years), high versus low KPS (<90 versus ≥90), absence of pain prior to SABR (pain versus no pain), GTV >37 cc and ≤37cc, interval between the end of chemotherapy and start of SABR (≤6 weeks versus >6 weeks after the last cycle of chemotherapy), and number of chemotherapy cycles (1-4 versus 5-8 versus >8 cycles).

Univariable and multivariable Cox proportional hazard analyses were performed to identify predictors for OS after SABR. Variables in these analyses included all aforementioned variables (see Kaplan-Meier analyses). Variables that were associated with OS at univariable analysis (p<0.2) were included in one single multivariable Cox proportional hazard model. Results of the Cox proportional hazard analyses are presented in hazard ratios (HR) with corresponding 95% confidence intervals (CI). A p-value lower than 0.05 was considered statistically significant. Backward selection was performed until the multivariable model comprised only significant parameters (i.e., *p*<0.05).

## Results

### Patient characteristics

Overall, 74 patients with unresectable LAPC who were initially treated with chemotherapy and subsequently with SABR were included. Median age was 66 years (range 36-81 years), 51.4% of patients were female. About half of patients had a KPS <90 (54.1%). Most patients had primary pancreatic head cancer (67.6%). The majority (87.8%) of patients received FOLFIRINOX, median number of cycles prior to SABR was 4. SABR was delivered with a median dose of 40 Gy (IQR 40-40) in 5 fractions within two weeks overall treatment time. In a single patient, treatment was stopped after 4 fractions because of grade 3 fatigue. In 4 patients an upfront decision was made to deliver 5 fractions of 7 Gy because of local ingrowth in the stomach or bowel (n=2) and because of tumor size (n=2). The delivered mean D2% (dose maximum) was 121% of the prescribed dose and mean GTV dose was 111% of the prescribed dose. Twenty-two patients received chemotherapy after treatment with SABR, mainly FOLFIRINOX (72.7%), ranging from 1 to 12 cycles. Patient characteristics are shown in **Table 1**.

**Table 1:** Baseline characteristics in patients with localized PDAC treated with chemotherapy and SABR.

Characteristic	Cohort (n=74)
Age, years, median (range)	66 (36-81)
Female sex, *n* (%)	38 (51)
Performance score (KPS), *n* (%) KPS <90 KPS 90-100	40 (54) 34 (46)
Tumor location*, n* (%) Head Body-tail	50 (68) 24 (32)
GTV, cc, median (range)	36.8 (7-117)
Radiation dose, Gy, median (IQR)(range)	40 (40-40)(32-40)
Number of fractions*	5
Induction chemotherapy, *n* (%) FOLFIRINOX Gemcitabine based	74 (100) 65 (88) 9 (12)
Number of cycles of induction chemotherapy, median (IQR) 1-4, *n* (%) 5-8, *n* (%) >8, *n* (%)	4 (4-8) 38 (51) 23 (31) 13 (18)
Chemotherapy post SABR, *n* (%)	22 (30)
FOLFIRINOX	16 (22)
Gemcitabine based	6 (8)
Number of cycles chemotherapy post SABR, 1-4, *n* (%) 5-8, *n* (%) >8, *n* (%)	12 (50) 6 (27) 4 (18)

* A single patient stopped after 4 fractions because of grade 3 fatigue.

### Survival outcomes

**Figure 1:**
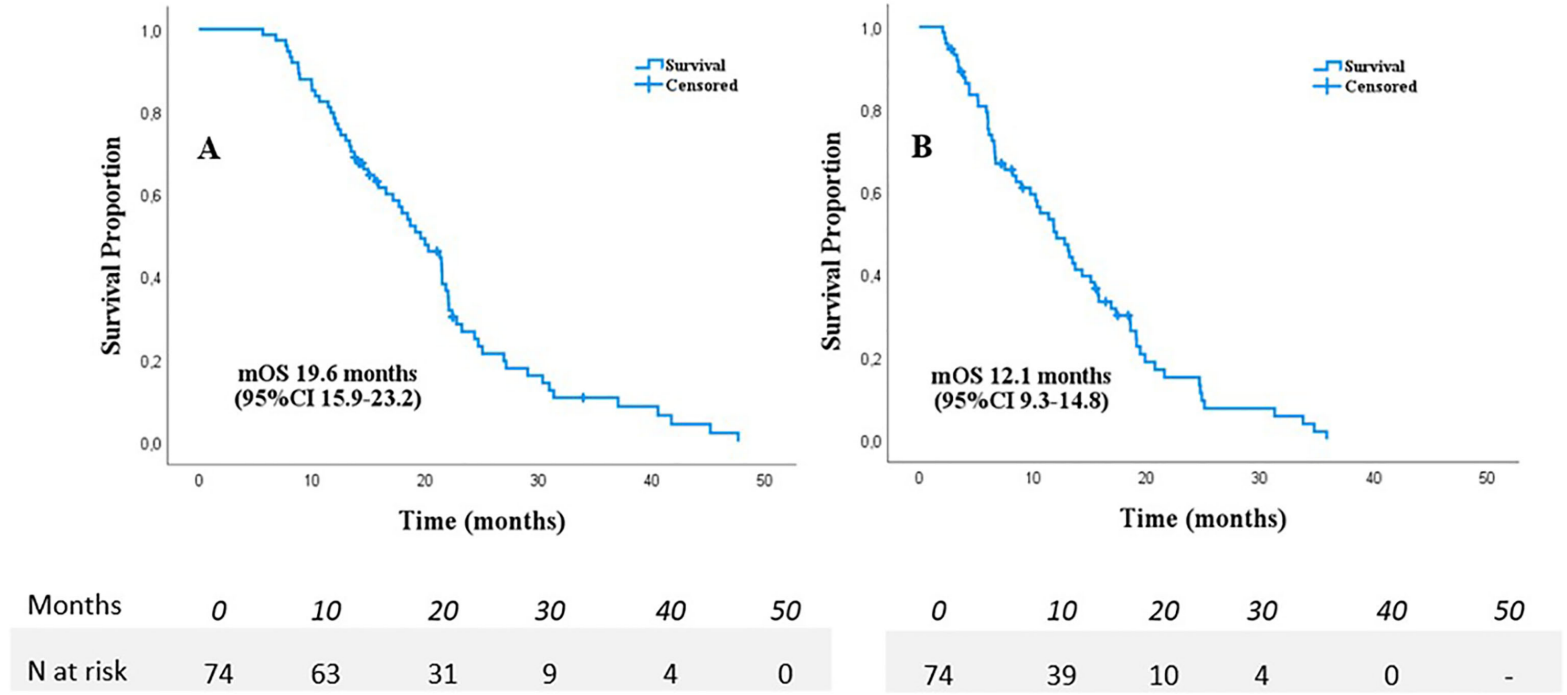
Overall survival from diagnosis **(A)** and start of SABR **(B)** in 74 patients with LAPC following chemotherapy.

Median follow-up time was 17.8 months from diagnosis and 10.5 months from SABR, no patients were lost to follow-up. A total of 63/74 (86.5%) patients died and 10 patients were censored for the survival analyses, only a single patient died following a non-pancreatic cancer related cause. Median OS from diagnosis was 19.6 months (95%CI 15.9-23.2 months) and 12.1 months (95%CI 9.3-14.8 months) from start of SABR (**Figure 1**). Kaplan-Meier analyses in separate subgroups revealed a better survival after start of SABR for patients with KPS ≥90 (17.3 versus 6.7 months, p<0.001), age ≤70 (15.4 versus 6.7 months p<0.001, >4 cycles of chemotherapy (13.2 versus 6.0 months, p<0.001), and absence of pain at the time of SABR (15.4 versus 7.6 months, p=0.010). Other tested variables did not affect OS in the Kaplan-Meier analyses, in particular also the time interval between induction chemotherapy and SABR was not significant (See **supplementary file**).

### Cox proportional hazard analyses

**Figure 2:**
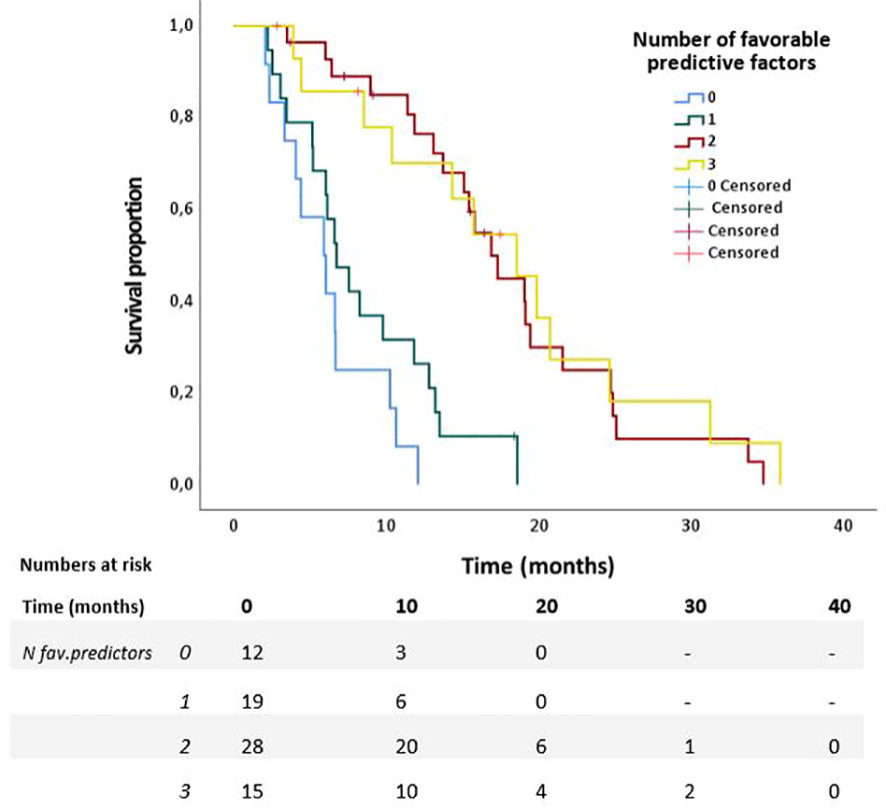
Overall survival of patients with 0 to 3 favorable predictors for survival from start of SABR in 74 patients with LAPC after chemotherapy.

**Figure 3:**
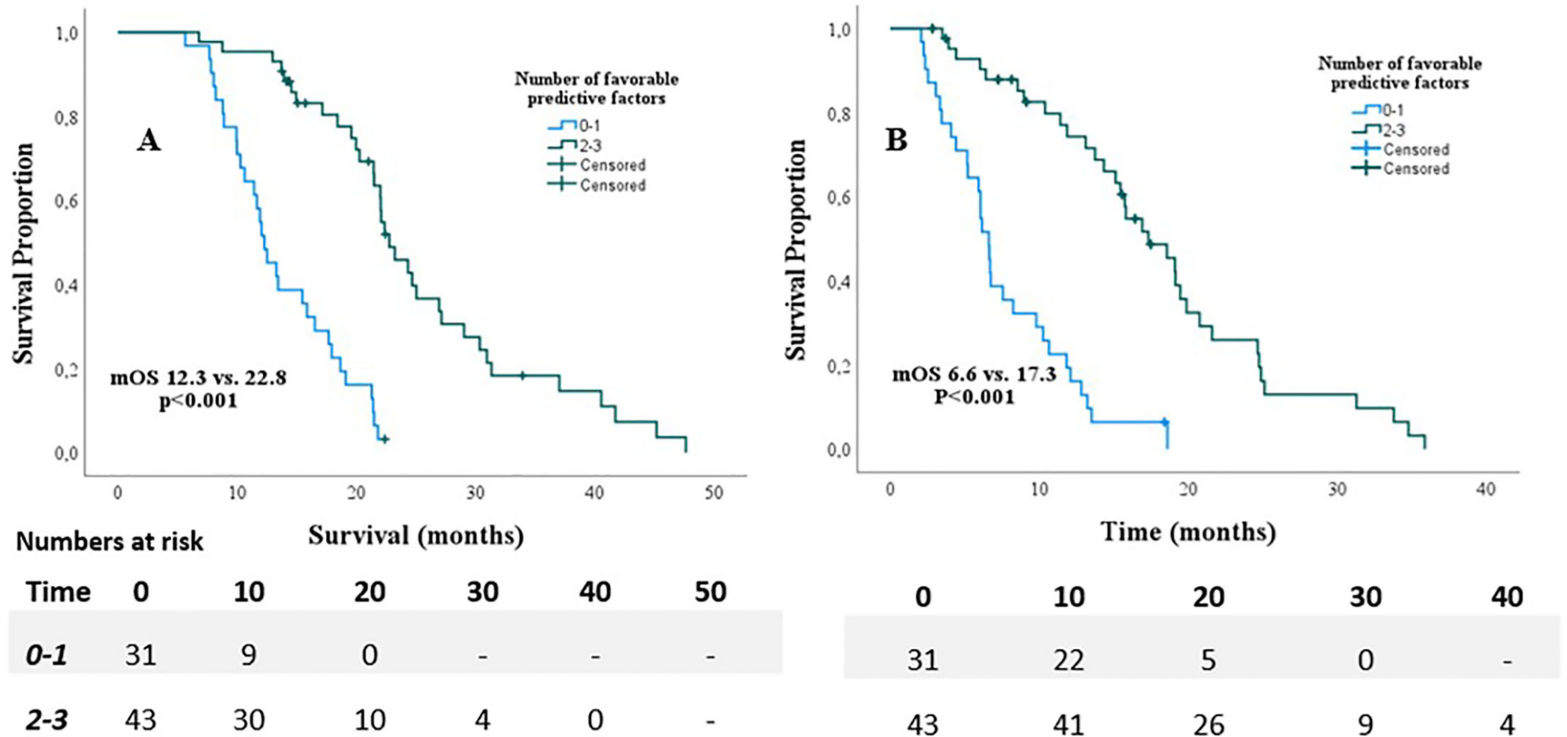
Overall survival from diagnosis **(A)** and start of SABR **(B)** for patients with <1 favorable predictor versus patients with 2 and 3 favorable predictors.

Univariable Cox regression analyses revealed that age ≤70, KPS≥90, absence of pain prior to SABR, Type of chemotherapeutic regimen, >4 cycles of chemotherapy, and <6 weeks interval between last cycle of chemotherapy and date of the first fraction, (p<0.2) (**Table 2**). These variables were therefore included in the multivariable model. This model revealed that age ≤70 (HR 0.42, p=0.007), KPS ≥90 (HR 0.49, p=0.026), and absence of pain prior to SABR (HR 0.40, p=0.001) were independently associated with improved OS. Results are shown in **Table 2**. In order to identify (un-)favorable patient groups, a cumulative score of the number of favorable predictive factors (KPS ≥90, age ≤70 and absence of pain) was generated, thus ranging from 0 to 3. Kaplan-Meier analysis showed a division between patients having no or one favorable factor versus more than one (**Figure 2**). An unfavorable (0-1 factor) and a favorable (2-3 factors) group were thus identified. Median survival from SABR for the unfavorable group was 6.6 months (N=31; 95%CI 5.9 – 7.3 months) versus 17.3 months (N=43; 95%CI 13.8 – 20.9 months) for the favorable group (**Figure 3**).

**Table 2:** Univariable and multivariable Cox regression analyses on predictors for overall survival in patients with localized PDAC treated with chemotherapy and SABR.

		Univariable			Multivariable			Backward selection
	*n*(%)	HR	95%CI	p-value	HR	95%CI	p-value	
Sex (male)	36 (49)	0.77	0.46-1.28	0.310				
Age ≤70 years	48 (65)	0.39	0.22-0.68	**0.001**	0.39	0.21-0.73	**0.003**	**0.001**
KPS 90-100	34 (46)	0.42	0.25-0.70	**<0.001**	0.44	0.24-0.81	**0.008**	**0.003**
Absence of pain*	38 (51)	0.52	0.32-0.87	**0.012**	0.40	0.23-0.70	**0.001**	**0.003**
Tumor location (head)	49 (66)	0.76	0.45-1.30	0.320				
Tumor Volume (GTV) >37cc	36 (49)	1.33	0.78-2.28	0.297				
Type of Chemotherapy*^	65 (88)	1.75	0.84-3.64	**0.138**	1.36	0.73-2.33	0.460	Removed step 1
*n* of cycles chemotherapy* 1-4 cycles (reference) 5-8 cycles >8 cycles	38 (51) 23 (31) 13 (18)	0.54 0.56	0.31-0.96 0.26-1.22	**0.076** 0.034 0.145	0.54 1.06	0.29-0.99 0.47-2.40	0.102	Removed step 3
Longer interval chemo-SABR^#^	24 (32)	1.44	0.84-2.48	**0.183**	1.31	0.73-2.33	0.366	Removed step 2

HR, hazard ratio; CI, confidence interval; KPS, Karnofsky performance score; GTV, Gross tumor Volume; cc.

*Prior to SABR.

^FOLFIRINOX (reference) (n=65) versus Gemcitabine based chemotherapy (n=9).

#Interval between last cycle of chemotherapy and date of first fraction is ≥6 weeks.

Bold values in the univariable column mean that they were considered significant to be incorporated in the multivariable analysis. The bold values in the multivariable column mean that they are significant.

### Progression of disease and local control rates

Six patients experienced a local recurrence. The actuarial local control rate at one year was 90.8%. Isolated loco-regional progression was observed in three patients (4.1%); the other three patients had a simultaneous diagnosis of distant metastases. Distant metastases without local recurrence were observed in 52 patients (70.3%), in the liver in 21 patients, peritoneum in 25 patients, and lungs in 17 patients. Sixteen patients had distant metastases in more than 1 site.

### Pain response

Prior to SABR, the distribution of abdominal pain was as follows: no pain in 51.4%, grade ≤2 in 47.2% and grade 3 in 1.4% of patients. Relief of pain was observed in 30 of 36 patients (83.3%) with pre-existing pain, either complete disappearance of complaints or allowing reduction of pain medication.

Pain indication prior to initial chemotherapy had a similar contribution compared to pain indicated prior to SABR: no pain in 45%, grade ≤2 in 53%, grade 3 in 0.0% and missing in 2% of patients.

### Toxicity

The only acute grade 3 toxicity that was observed was fatigue in two patients (2.7%); no grade 3 nausea, vomiting or diarrhea was seen. A transient increase or occurrence of abdominal pain was observed in 13/74 patients (17.6%) during or in the first weeks after treatment. This increase in pain did not exceed grade 2. Two patients (2.7%) experienced late grade 3 toxicity including one patient with gastrointestinal bleeding (1.4%) and one patient with suspicion of gastrointestinal obstruction (1.4%). No patient experienced late grade 3 pain, nausea, fatigue, or diarrhea.

## Discussion

The role of radiotherapy in the treatment of LAPC is under debate in the current international guidelines. Two randomized trials in patients with LAPC comparing gemcitabine monotherapy with gemcitabine plus conventionally fractionated radiotherapy (CFRT) reported contradictory results with respect to OS (17, 18). Both trials were conducted in an era in which gemcitabine-based systemic therapy was predominantly administered, whereas currently FOLFIRINOX is, like in 88% of our (fitter) patients (5, 7). Gemcitabine-based regimens, with or without conventional radiation, have long been the standard of care, resulting in a median OS of 9–11 months in patients with LAPC (18, 19). The use of FOLFIRINOX chemotherapy has improved survival; however, the prognosis for patients with locally advanced pancreatic cancer remains poor, with a median OS of 12–14 months (18, 20). As current systemic treatment becomes more efficient, optimization of local control is increasingly important. With no randomized studies available to compare the efficacy and toxicity of CFRT and SABR in LAPC patients, an extensive systematic review and meta-analysis was performed recently by Tchelebi et al. (21) This study suggest that SABR may offer a modest improvement in 2-year OS (26.9% vs 13.7%) in combination with a favorable acute toxicity profile (5.6% vs 37.7%).

A retrospective cohort demonstrated improved loco-regional control in patients with LAPC who could be treated with a simultaneous integrated boost up to a biologically effective dose (BED_10_) of more than 70 Gy (22). This dose-escalation was only feasible in a quarter of the patients in whom the tumor was at more than 1 cm distance from the closest gastrointestinal mucosa. With local radiation dose-escalation in mind, the application of SABR may be a more promising approach. A systematic literature review of SABR for LAPC including more than 1000 patients showed a local control rate at one year of 72.3% (12). Although recent series suggest that further dose-escalation, e.g. using MR-guided radiotherapy as performed in this series, may allow for BED_10_ of more than 100 Gy_10,_ we observed a local control rate of 90% with a BED_10_ of 72 Gy_10_ in five fractions (23, 24). Two similar retrospective studies included 149 and 62 patients also observed a high local control rates of respectively 86.0% and 87.9% at one year after SABR, and warrant the need for prospective evaluation (25, 26). The potential advantages and workflow of SABR performed as non-invasive MR-guided radiotherapy are outside the scope of this paper, but have been described previously (27, 28).

The findings of the present study should be interpreted in light of some limitations. First, a heterogeneous study population was included with possible selection bias due to the non-randomized single-arm design of this study. Differences in indication (primary LAPC and inoperable PC), use of chemotherapeutic regimen, and number of cycles of induction chemotherapy existed, however the present study did not find a prognostic relevance for the latter parameters. In addition, patients with higher age, larger tumors, and worse KPS were included, thus providing the possibility to compare outcomes in these subgroups. Moreover, by including a heterogeneous group treated with to a very similar/homogenous treatment with regard to dose, number of fractions and chemotherapeutic regimen, more can be concluded about the different clinical parameters. Second, it is desirable to find objective, rather than subjective, parameters to be incorporated in prediction models. However, in the present study absence of pain appeared to be a better predictive factor for survival compared to GTV (regardless incorporation of GTV as a continuous or categorized variable). No significant correlation was found between pain and GTV, hereafter both variables were included in the statistical analysis. Third, we were not able to include the value serum CA19-9 in our regression model due to a high proportion of invalid CA19-9 values. Given that CA19-9 levels and its reduction influence OS in PDAC, it would be interesting to find out if CA19-9 impacts survival and should be incorporated in patient selection for SABR (29, 30). Especially given that a high proportion of patients in the present study developed distant metastasis. Last, we were not able to compare these findings to a similar group treated with systemic therapy only, which is yet to be evaluated by a new, but still pending trial in unresectable LAPC comparing standard of care versus standard of care with SABR in unresectable PDAC (LAPSTAR), as well as pending trials comparing (m)FOLFIRINOX with or without additional SABR in the treatment of LAPC (NCT01827553 and NCT04986930).

The present study analyzed the outcomes in patients with LAPC, uniformly treated with initial chemotherapy followed by ablative SABR. Consistent with prior publications, SABR was well tolerated with low rates of acute and late toxicity, (<3%). The OS of 19.6 months is encouraging, but also underscores the need for clear clinical parameters to identify patients who may benefit from local ablative therapy following chemotherapy. The prognostic factors found, i.e. good performance, age younger than 70 years, and absence of pain, showing a substantial and relevant impact on survival in LAPC patients with almost one year median survival difference between the favorable and unfavorable groups. One issue to be addressed is the question whether the unfavorable group should be treated with SABR. The positive response to pain in 83% of patients, confirms the palliative effect of radiotherapy in prior studies, but palliative conventional RT may be sufficient (31, 32).

## Conclusion

The impact of clinical parameters on survival of patients with LAPC after chemotherapy is considerable and should be taken into account in the selection for subsequent SABR. The value of SABR as local ablative therapy following chemotherapy should be investigated in randomized controlled trials, and patient performance status, age, and absence of pain should be taken into account in the design of such trial.

## Data availability statement

The raw data supporting the conclusions of this article will be made available by the authors, upon reasonable request.

## Ethics statement

Ethical review and approval was not required for the study on human participants in accordance with the local legislation and institutional requirements. Written informed consent for participation was not required for this study in accordance with the national legislation and the institutional requirements.

## Author contributions

Conceptualization, and design of the study: AB, FL, DD. AB, FL, DD have organized the database, performed the statistical analysis with the help of SD, and wrote the first draft of the manuscript. AB, FL, DD, SD, MM, EV, MB, GT, JV, BS, JW and GK wrote sections of the manuscript and revised the manuscript, all contributed equally. All authors have read and agreed to the published version of the manuscript.
