# Supplementary material for: Optimizing patient selection for stereotactic ablative radiotherapy in patients with locally advanced pancreatic cancer after initial chemotherapy - a single center prospective cohort
Source: Front Oncol. 2023 May 31;13:1149961. doi: 10.3389/fonc.2023.1149961 (PMC10264658; doi:10.3389/fonc.2023.1149961)

**Optimizing patient selection for stereotactic ablative radiotherapy in patients with locally advanced pancreatic cancer after initial chemotherapy - A single center prospective cohort**

*D. Doppenberg MD ^1,2,3^, F.J. Lagerwaard MD PhD^1,2^, S, van Dieren, Msc, Ph ^3^, M.R. Meijerink MD PhD^2,4^, J.J. van der Vliet MD PhD^2,5,6^, M.G. Besselink MD MSc PhD ^2,3^, G. van Tienhoven MD PhD ^1,2^, E. Versteijne MD PhD ^1,2^, B.J Slotman MD PhD^1,2^, J.W. Wilmink MD PhD^2,7^, Geert Kazemier MD PhD^2,8^, A.M.E. Bruynzeel MD PhD^1,2*^*

^1^Amsterdam UMC, location Vrije Universiteit Amsterdam, Department of Radiation Oncology, Amsterdam, the Netherlands

^2^Cancer Center Amsterdam, the Netherlands

^3^Amsterdam UMC, location University of Amsterdam, Department of Surgery, Amsterdam, the Netherlands

^4^Amsterdam UMC, location Vrije Universiteit Amsterdam, Department Intervention Radiology, Amsterdam, the Netherlands

^5^Amsterdam UMC, location Vrije Universiteit Amsterdam, Department of Medical Oncology, Amsterdam, the Netherlands

^6^ LAVA Therapeutics, Utrecht, the Netherlands

^7^Amsterdam UMC, location University of Amsterdam Amsterdam, Department of Medical Oncology, Amsterdam, the Netherlands

^8^Amsterdam UMC, location Vrije Universiteit Amsterdam, Department of Surgery, Amsterdam, the Netherlands

**Corresponding author**

A.M.E. Bruynzeel, MD, PhD

Amsterdam UMC, location Vrije Universiteit Amsterdam, Department of Radiation Oncology, Cancer center Amsterdam, Amsterdam UMC

De Boelelaan 1117, Amsterdam, The Netherlands

Tel: +31 20 444 4444

Mail: ame.bruynzeel@amsterdamumc.nl

**Index:**

| Title | Page |
| --- | --- |
| S1. Overall survival from start of SABR in 74 patients with LAPC following chemotherapy, female versus male sex | 3 |
| S2. Overall survival from start of SABR in 74 patients with LAPC following chemotherapy, age ≤70 versus >70 years | 3 |
| S3. Overall survival from start of SABR in 74 patients with LAPC following chemotherapy, KPS 90-100 versus <90 | 4 |
| S4. Overall survival from start of SABR in 74 patients with LAPC following chemotherapy based on tumor location in the pancreas: Head versus corpus/tail | 4 |
| S5. Overall survival from start of SABR in 74 patients with LAPC following chemotherapy, absence of pain versus pain prior to treatment | 5 |
| S6. Overall survival from start of SABR in 74 patients with LAPC following chemotherapy, GTV ≤37cc versus >37cc | 5 |
| S7. Overall survival from start of SABR in 74 patients with LAPC following chemotherapy, 1-4 versus 5-8 versus >8 cycles of induction chemotherapy | 6 |
| S8. Overall survival from start of SABR in 74 patients with LAPC following chemotherapy, interval between induction chemotherapy and start SABR <6 weeks versus ≥6 weeks | 6 |

**S1. Overall survival from start of SABR in 74 patients with LAPC following chemotherapy, female versus male sex**


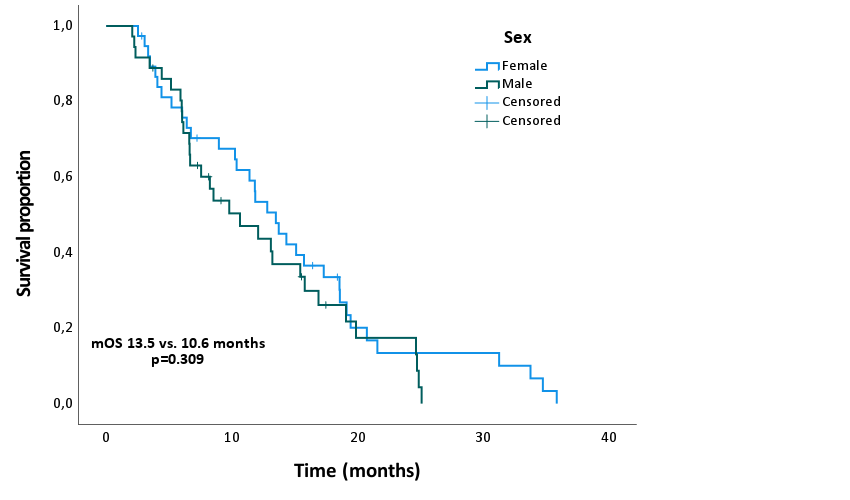


**S2. Overall survival from start of SABR in 74 patients with LAPC following chemotherapy, age ≤70 versus >70 years**


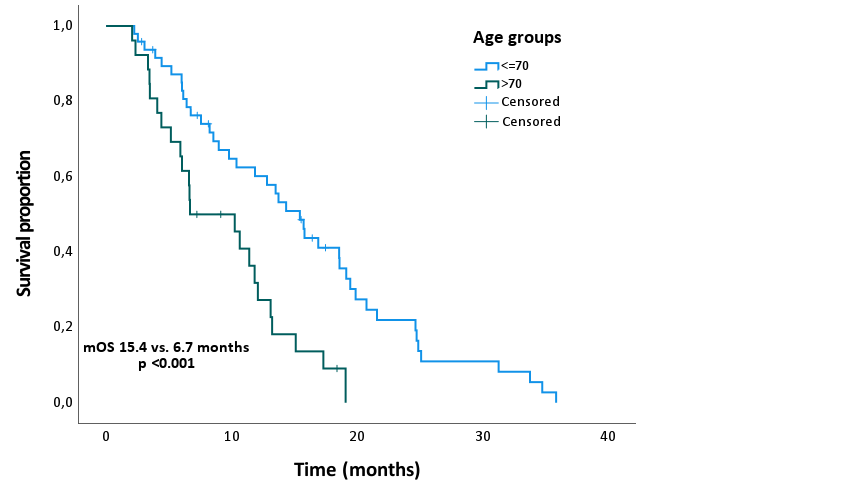


**S3. Overall survival from start of SABR in 74 patients with LAPC following chemotherapy, KPS 90-100 versus <90**
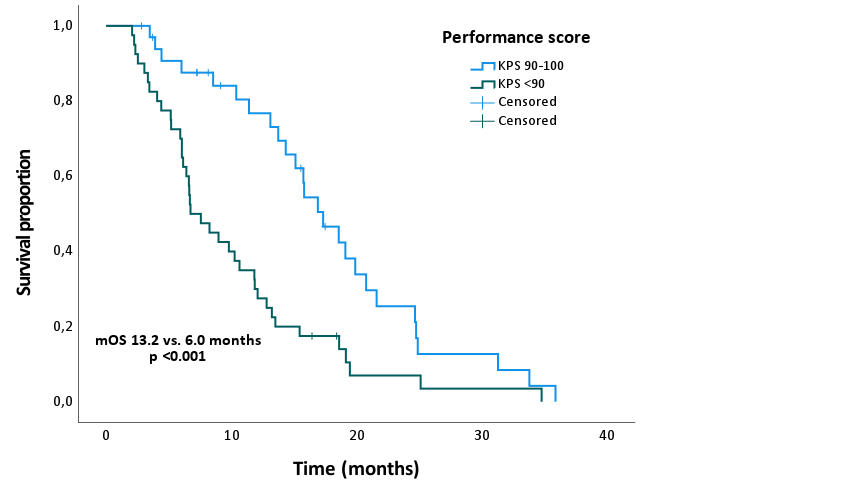


**S4. Overall survival from start of SABR in 74 patients with LAPC following chemotherapy based on tumor location in the pancreas: Head versus corpus/tail**


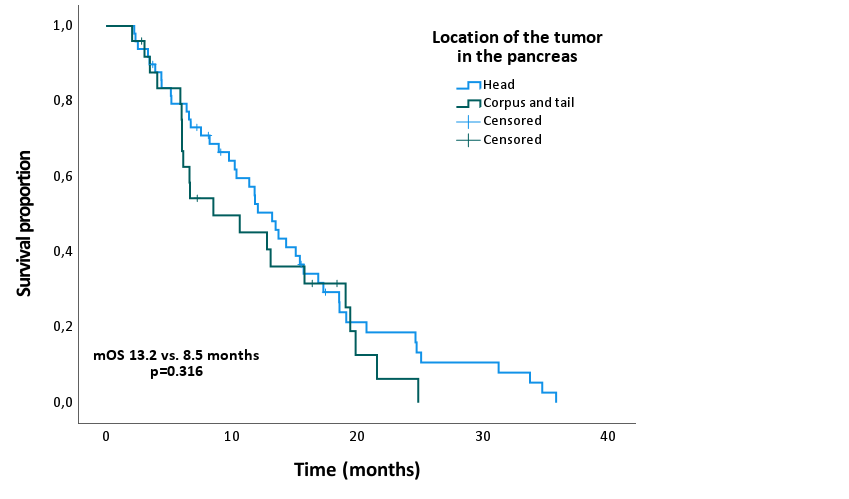


**S5. Overall survival from start of SABR in 74 patients with LAPC following chemotherapy, absence of pain versus pain prior to treatment**


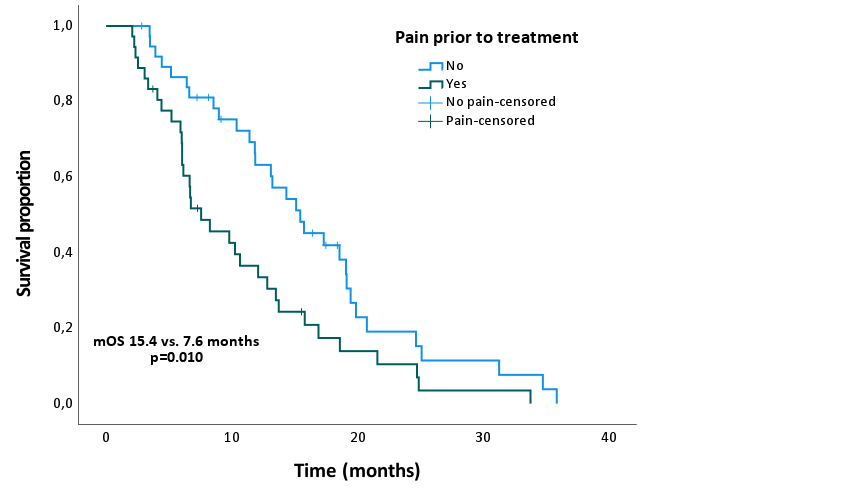


**S6. Overall survival from start of SABR in 74 patients with LAPC following chemotherapy, GTV ≤37cc versus >37cc**


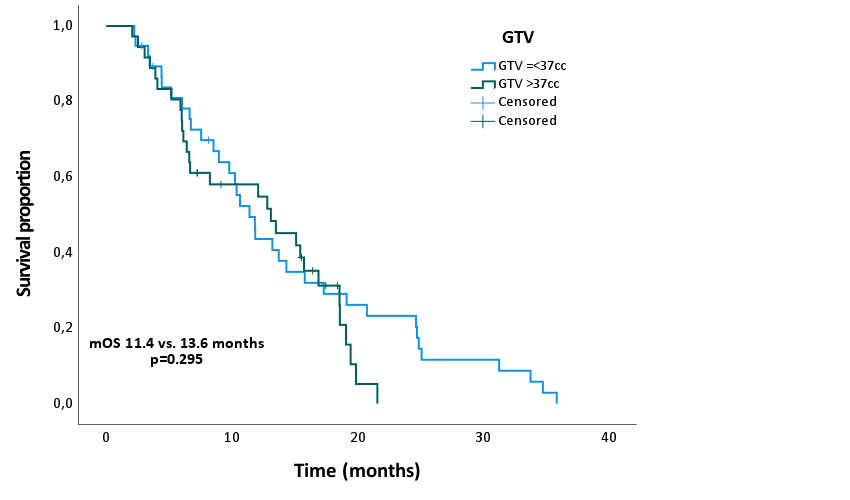


**S7. Overall survival from start of SABR in 74 patients with LAPC following chemotherapy, 1-4 versus 5-8 versus >8 cycles of induction chemotherapy**


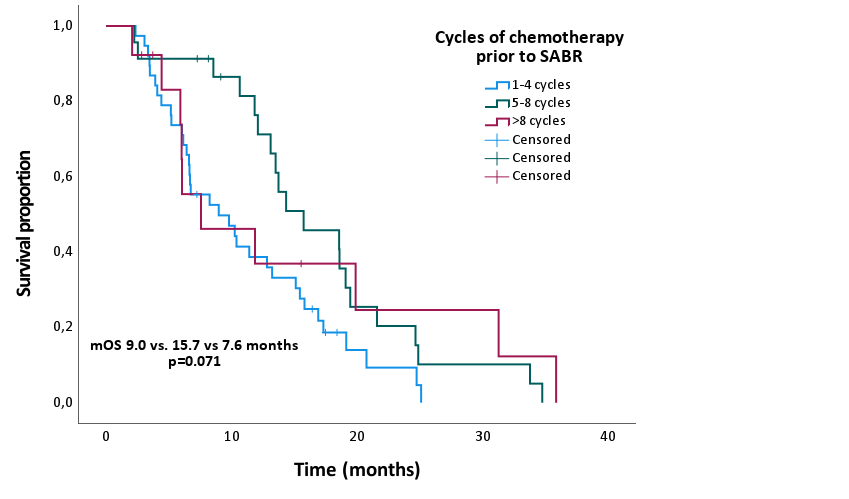


**S8. Overall survival from start of SABR in 74 patients with LAPC following chemotherapy, interval between induction chemotherapy and start SABR <6 weeks versus ≥6 weeks**


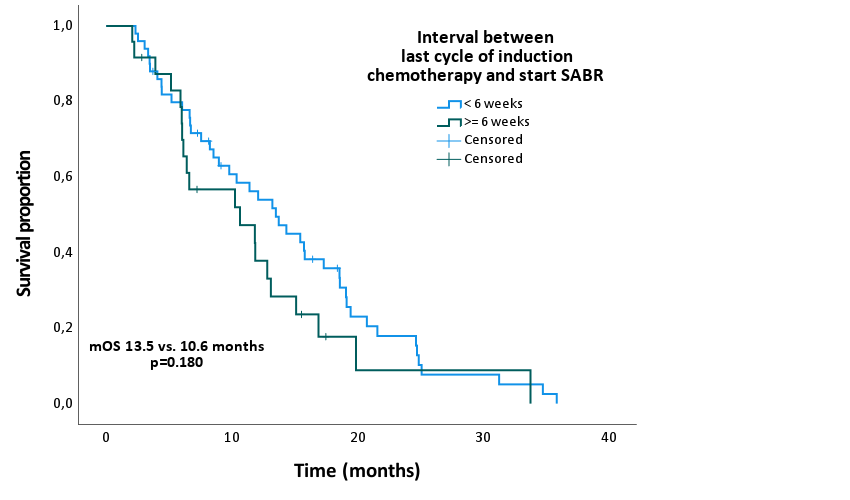

Supplement: Supplementary file 1 [file Table_1.docx]
